# Supplementary figures and images for: Discovery of a potent, selective, and tumor-suppressing antibody antagonist of adenosine A2A receptor
Source: PLoS One. 2024 Jun 5;19(6):e0301223. doi: 10.1371/journal.pone.0301223 (PMC11152298; doi:10.1371/journal.pone.0301223)

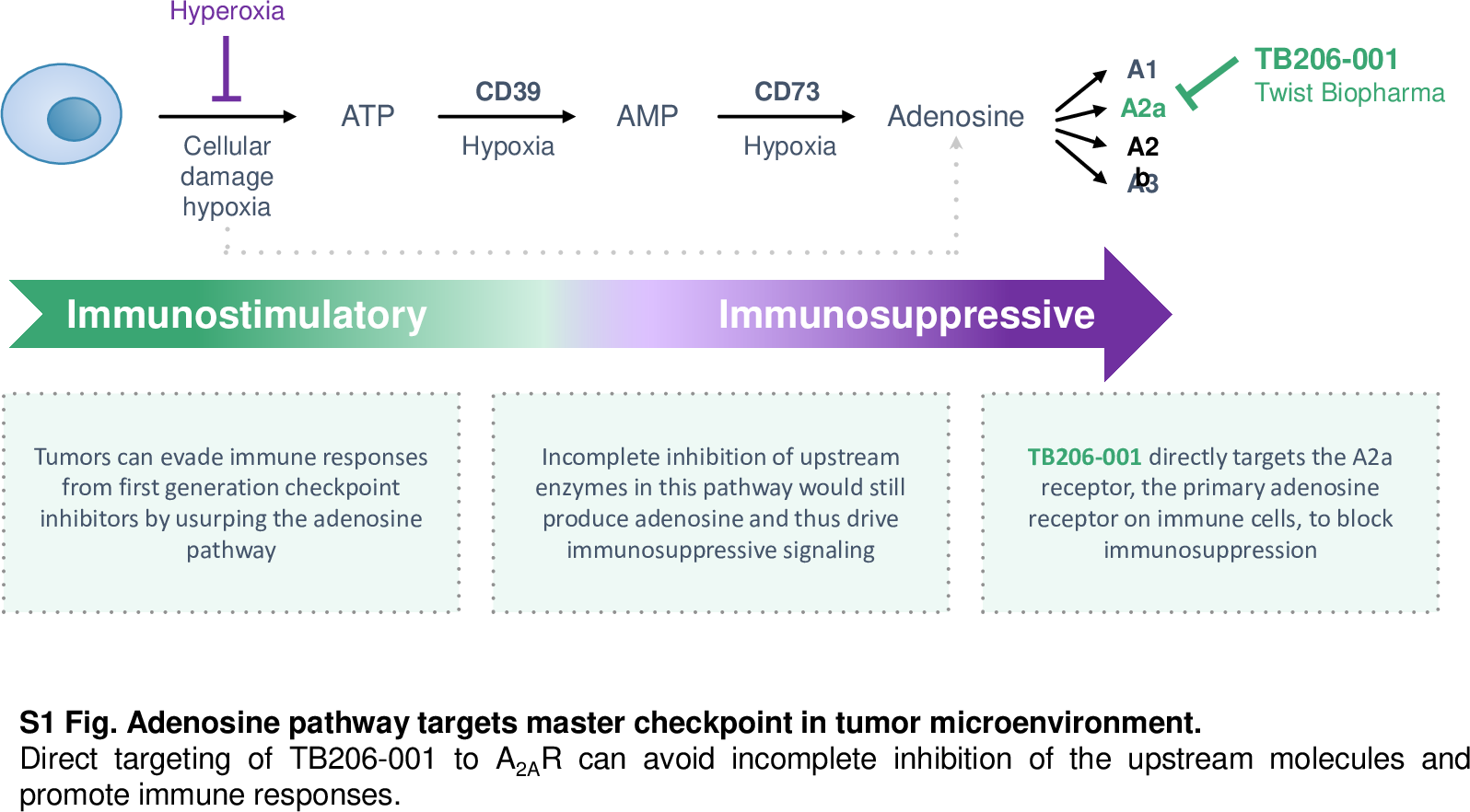

Supplement: S1 Fig — Direct targeting of TB206-001 to A2AR can avoid incomplete inhibition of the upstream molecules and promote immune responses. (TIF) [file pone.0301223.s001.tif]
