# Supplementary material for: Discovery of a potent, selective, and tumor-suppressing antibody antagonist of adenosine A2A receptor
Source: PLoS One. 2024 Jun 5;19(6):e0301223. doi: 10.1371/journal.pone.0301223 (PMC11152298; doi:10.1371/journal.pone.0301223)
Supplement: S1 Table — (DOCX) [file pone.0301223.s002.docx]

**Supporting information**

**S1 Table. Raw data of Fig 2.**

**Raw data of Fig 2A.** TB206-001 binds to hA_2A_R-overexpressing HEK293 cells.

|  | Readout: MFI |  |
| --- | --- | --- |
| Antibody concentration (nM) | TB206-001 (hIgG) | Control A2a (mIgG) |
| 100 | 350976 | 395890 |
| 33.33333333 | 328543 | 319636 |
| 11.11111111 | 282903 | 203682 |
| 3.703703704 | 110536 | 163795 |
| 1.234567901 | 62684 | 62347 |
| 0.411522634 | 52578 | 59063 |
| 0.137174211 | 21368 | 39369 |
| 0.045724737 | 12423 | 21646 |

**Raw data of Fig 2B.** TB206-001 cross-reacts with mA_2A_R but not hA_1_R, hA_2B_R, and hA_3_R. Standard deviations are generated by Prism Graphpad.

|  | Readout: MFI | |  |  |  |  |  |  |  |  |  |  |
| --- | --- | --- | --- | --- | --- | --- | --- | --- | --- | --- | --- | --- |
|  | hA1 | | | hA2b | | | hA3 | | | mA2a | | |
| Cell only | 28.9 | 36.2 |  | 36.2 | 36.2 |  | 36.2 | 28.9 |  | 36.2 | 36.2 |  |
| Seceondary antibody only | 28.9 | 36.2 |  | 36.2 | 43.4 |  | 36.2 | 28.9 |  | 43.4 | 36.2 |  |
| Positive control antibodies | 6913 | 6300 | 7300 | 4122 | 3700 | 4500 | 3739 | 3200 | 4500 | 8731 | 8300 | 8800 |
| TB206-001 | 994 | 700 | 1110 | 1046 | 700 | 1110 | 2198 | 1800 | 2300 | 7161 | 7300 | 6690 |

**Raw data of Fig 2C.** TB206-001 cross-reacts with cynomolgus PBMCs. Standard deviations are generated by Prism Graphpad.

| Readout: MFI |  |  |
| --- | --- | --- |
| TB206-001 | 2Ab staining | Cell only |
| 2163 | 182 | 161 |
| 1800 | 170 | 170 |
| 2340 |  |  |
