# Supplementary material for: Discovery of a potent, selective, and tumor-suppressing antibody antagonist of adenosine A2A receptor
Source: PLoS One. 2024 Jun 5;19(6):e0301223. doi: 10.1371/journal.pone.0301223 (PMC11152298; doi:10.1371/journal.pone.0301223)
Supplement: S2 Table — (DOCX) [file pone.0301223.s003.docx]

**Supporting information**

**S2 Table. Raw data of Fig 3.** Standard deviations, graphs, and IC50 are obtained from Prism Graphpad.

**Raw data of Fig 3A.** Detection of cAMP in HEK293 cells that overexpress hA_2A_R. TB206-001 dose-dependently increased the RFU ratio (665/615 nm).

| Readout: RFU ratio 665/615nm | | |
| --- | --- | --- |
| Antibody concentration (nM) | TB206-001 | |
| 100 | 0.242296 | 0.231468 |
| 33.3333333 | 0.227338 | 0.211292 |
| 11.1111111 | 0.218028 | 0.202425 |
| 3.7037037 | 0.212138 | 0.192098 |
| 1.2345679 | 0.201468 | 0.182465 |
| 0.41152263 | 0.194363 | 0.177456 |
| 0.13717421 | 0.187634 | 0.172927 |
| 0.04572474 | 0.157656 | 0.165768 |

**Raw data of Fig 3B.** TB206-001 antagonized NECA-stimulated IFN-γ release in T cell-activated (CD3/CD28-simulated) PBMCs.

|  | Readout: IFNg (pg/ml) | |  |  |
| --- | --- | --- | --- | --- |
| Antibody concentration (nM) | TB206-001 | | ZM-241385 | |
| 100 | 1287 | 1402.6 | 1487.3 | 1334.3 |
| 33.3333333 | 1170.7 | 1366.4 | 1085.6 | 1281.4 |
| 11.1111111 | 811.3 | 1090.2 | 506.5 | 852.6 |
| 3.7037037 | 529.8 | 781.8 | 379.9 | 572.2 |
| 1.2345679 | 425.8 | 736.1 | 169.4 | 491.9 |
| 0.41152263 | 224.9 | 330.4 | 166.3 | 213.9 |
| 0.13717421 | 143.8 | 308.8 | 165.6 | 190.7 |

**Raw data of Fig 3C.** Effect of NECA ligand concentration on TB206-001 antagonism of NECA-stimulated IFN-γ release.

|  | Readout: IFNg (pg/ml) | |  |  |  |  |
| --- | --- | --- | --- | --- | --- | --- |
| TB206-1 (nM) | NECA (10uM) | | NECA (3uM) | | NECA (1uM) | |
| 100 | 866.6 | 970.7 | 1295.2 | 927.7 | 1508.6 | 1480.7 |
| 33.3333333 | 782.5 | 820 | 933.9 | 988.4 | 1486.8 | 1387.6 |
| 11.1111111 | 649.4 | 682.8 | 833.2 | 728.2 | 1337.7 | 1319.9 |
| 3.7037037 | 642.4 | 555.1 | 518.3 | 415 | 993.3 | 813.3 |
| 1.2345679 | 374.3 | 411.4 | 257.7 | 306.2 | 791.6 | 675.6 |
| 0.41152263 | 357.9 | 311 | 250.8 | 243.7 | 574.7 | 428.6 |
| 0.13717421 | 299.7 | 292.7 | 249 | 226.6 | 339.6 | 208.8 |
| 0.04572474 | 288.6 | 244.1 | 235.9 | 205.7 | 221.4 | 202 |

**Raw data of Fig 3D.** Isolated T cells activation is assessed by cell proliferation.

|  | T cell proliferation Fold increase | | |  |
| --- | --- | --- | --- | --- |
| Antibody (nM) | TB206-001 | | AB928 | |
| 100 | 4.4 | 4.88888889 | 4.2 | 4.66666667 |
| 33.3333333 | 3.3 | 3.66666667 | 3.2 | 3.55555556 |
| 11.1111111 | 3 | 3.33333333 | 2.3 | 2.55555556 |
| 3.7037037 | 2.2 | 2.44444444 | 1.9 | 2.11111111 |
| 1.2345679 | 1.8 | 2 | 1 | 1.11111111 |
| 0.41152263 | 1.6 | 1.77777778 | 0.8 | 0.88888889 |
| 0.13717421 | 1 | 1.11111111 | 0.7 | 0.77777778 |
| 0.04572474 | 1 | 1.11111111 | 0.5 | 0.88888889 |

**Raw data of Fig 3E.** Isolated T cells activation is assessed by up-regulation of activation marker CD25.

|  | CD25+ cell (%) | |  |  |
| --- | --- | --- | --- | --- |
| Antibody (nM) | TB206-001 | | AB928 | |
| 100 | 20.4 | 25 | 15 | 22 |
| 33.3333333 | 13.1 | 23 | 12.5 | 19 |
| 11.1111111 | 12.2 | 19 | 12.5 | 17 |
| 3.7037037 | 11.7 | 18 | 12.1 | 15 |
| 1.2345679 | 11.4 | 16 | 10.6 | 15 |
| 0.41152263 | 11.2 | 12 | 10.2 | 13 |
| 0.13717421 | 10.6 | 14 | 9.78 | 10 |
| 0.04572474 | 9.49 | 10 | 6.9 | 8 |
| 0.01524158 | 8 | 9 | 5 | 7 |
| 0.00508053 | 5 | 8 | 4 | 6 |

**Raw data of Fig 3F.** Isolated NK cell activation is detected by IFN-γ release.

|  | Readout: IFNg (pg/ml) |  |  |  |
| --- | --- | --- | --- | --- |
| Antibody (nM) | TB206-001 | | AB928 | |
| 100 | 154.25 | 201.375 | 135.625 | 149 |
| 33.3333333 | 237.5 | 245.875 | 155.75 | 146.625 |
| 11.1111111 | 148.125 | 216.375 | 134.875 | 125.125 |
| 3.7037037 | 242.625 | 184 | 213.375 | 161.25 |
| 1.2345679 | 163.375 | 159.125 | 283.125 | 176.75 |
| 0.41152263 | 221.75 | 178 | 226.25 | 156.25 |
| 0.13717421 | 167.625 | 266.5 | 196.5 | 158 |
| 0.04572474 | 219.5 | 213.125 | 201.125 | 205.875 |

**Raw data of Fig 3G.** Isolated NK cell activation is detected by activation marker CD107.

| CD107+ cell (%) | |
| --- | --- |
| Untreated | IL15 |
| 0.85 | 15.2 |
| 0.9 | 20.8 |
