# Supplementary material for: Discovery of a potent, selective, and tumor-suppressing antibody antagonist of adenosine A2A receptor
Source: PLoS One. 2024 Jun 5;19(6):e0301223. doi: 10.1371/journal.pone.0301223 (PMC11152298; doi:10.1371/journal.pone.0301223)
Supplement: S3 Table — (DOCX) [file pone.0301223.s004.docx]

**Supporting information**

**S3 Table. Raw data of Fig 4.** Standard deviations, graphs, IC50, and analytical significant are obtained from Prism Graphpad.

**Raw data of Fig 4B.** TB206-001-IgG1, pembrolizumab, and the small molecule AZD4635 suppressed the growth of COLO 205 tumors in HuCD34NCG mice.

| Study Days | Isotype control | | | Human Anti-PD1 | | | TB206-001 | | | AZD4635 | | |
| --- | --- | --- | --- | --- | --- | --- | --- | --- | --- | --- | --- | --- |
|  | Tumor volume (mm3) | Standard deviation | Number of mice | Tumor volume (mm3) | Standard deviation | Number of mice | Tumor volume (mm3) | Standard deviation | Number of mice | Tumor volume (mm3) | Standard deviation | Number of mice |
| 0 | 108.06 | 5.12 | 6 | 107.74 | 7.2 | 6 | 108.1 | 7.35 | 6 | 106.67 | 7.11 | 6 |
| 3 | 191.29 | 15.98 | 6 | 156.11 | 15.55 | 6 | 162.38 | 21.54 | 6 | 163.99 | 11.01 | 6 |
| 5 | 238.65 | 19.21 | 6 | 205.83 | 22.78 | 6 | 199.43 | 33.64 | 6 | 201.19 | 10.12 | 6 |
| 9 | 344.26 | 45.54 | 6 | 292.57 | 31.96 | 6 | 281.55 | 38.82 | 6 | 260.81 | 40.94 | 5 |
| 12 | 468.7 | 60.5 | 6 | 398.32 | 32.58 | 6 | 368.16 | 56.35 | 6 | 281.55 | 33.59 | 5 |
| 15 | 614.88 | 92.41 | 6 | 480.22 | 45.08 | 6 | 457.87 | 60.09 | 6 | 311.02 | 34.54 | 5 |
| 18 | 819.87 | 124.12 | 6 | 587.62 | 59.86 | 6 | 511.89 | 63.64 | 6 | 379.81 | 49.89 | 5 |
| 22 | 1012.5 | 157.8 | 6 | 747.97 | 72.12 | 6 | 658.53 | 88.25 | 6 | 476.56 | 49.52 | 5 |
| 24 | 1132.15 | 186.91 | 6 | 856.6 | 86.71 | 6 | 734.35 | 98.79 | 6 | 557.13 | 68.1 | 5 |

**Raw data of Fig 4C.** TB206-001-IgG4 binds to hA_2A_R-overexpressing HEK293 cells

|  | Readout: MFI | |
| --- | --- | --- |
| Antibody concentration (nM) | A2a-HEK | HEK |
| 100 | 2952 | 113 |
| 33.33 | 1833 | 102 |
| 11.11 | 461 | 101 |
| 3.704 | 330 | 109 |
| 1.235 | 307 | 105 |
| 0.412 | 210 | 93.9 |
| 0.137 | 199 | 64 |
| 0.046 | 191 | 61.9 |

**Raw data of Fig 4D.** TB206-001-IgG4 promotes IFN-γ release from NECA-stimulated, T cell-activated PBMCs.

|  | Readout: IFNg (pg/ml) | |  |  |
| --- | --- | --- | --- | --- |
| Antibody concentration (nM) | TB206-001-IgG1 | | TB206-001-IgG4 | |
| 100 | 1508.6 | 1480.7 | 1758 | 1892.8 |
| 33.33 | 1486.8 | 1387.6 | 1716 | 1706.5 |
| 11.11 | 1337.7 | 1319.9 | 1482 | 1600.5 |
| 3.704 | 993.3 | 813.3 | 1306 | 1322.6 |
| 1.235 | 791.6 | 675.6 | 1276 | 1304.7 |
| 0.412 | 574.7 | 428.6 | 768.7 | 1172.8 |
| 0.137 | 339.6 | 208.8 | 906.5 | 560.9 |
| 0.046 | 221.4 | 202 | 318 | 382.5 |

**Raw data of Fig 3E.** TB206-001-IgG4 and TB206-001-IgG1 suppressed the growth of COLO 205 tumors in HuCD34NCG mice with similar potency.

| Study Days | Isotype control | | | TB206-001-IgG1 | | | TB206-001-IgG4 | | |
| --- | --- | --- | --- | --- | --- | --- | --- | --- | --- |
|  | Tumor volume (mm3) | Standard deviation | Number of mice | Tumor volume (mm3) | Standard deviation | Number of mice | Tumor volume (mm3) | Standard deviation | Number of mice |
| 0 | 104.26 | 14.31 | 6 | 101.71 | 7.78 | 6 | 105.16 | 13.16 | 6 |
| 2 | 196.18 | 29.96 | 6 | 201.82 | 22.11 | 6 | 175.41 | 18.52 | 6 |
| 5 | 282.5 | 46.27 | 6 | 331.8 | 45.04 | 6 | 269.99 | 31.3 | 6 |
| 7 | 379.49 | 44.08 | 6 | 440.22 | 62.23 | 6 | 342.62 | 48.68 | 6 |
| 9 | 531.49 | 53.73 | 6 | 530.98 | 70.83 | 6 | 437.94 | 65.54 | 6 |
| 12 | 678.46 | 67.26 | 6 | 445.73 | 63.98 | 6 | 501.18 | 108.27 | 6 |
| 14 | 768.42 | 71.46 | 6 | 540.7 | 86.65 | 6 | 578.92 | 115.47 | 6 |
| 16 | 841.4 | 77.17 | 6 | 661.42 | 93.61 | 6 | 693.27 | 121.18 | 6 |
| 19 | 1077.33 | 110.89 | 6 | 860.14 | 112.81 | 6 | 907.86 | 105.4 | 6 |
| 21 | 1249.13 | 139.15 | 6 | 955.02 | 123.16 | 6 | 1008.67 | 135.83 | 6 |
| 23 | 1324.12 | 147.39 | 6 | 1058.47 | 170.01 | 5 | 1101.33 | 134.55 | 6 |
| 26 | 1595.07 | 227.6 | 6 | 1394.9 | 278.49 | 5 | 1208.35 | 145.51 | 6 |
