# Supplementary material for: Discovery of a potent, selective, and tumor-suppressing antibody antagonist of adenosine A2A receptor
Source: PLoS One. 2024 Jun 5;19(6):e0301223. doi: 10.1371/journal.pone.0301223 (PMC11152298; doi:10.1371/journal.pone.0301223)
Supplement: Attachment — Submitted filename: Comments_AK.docx [file pone.0301223.s005.docx]

In the present manuscript, the author screened and discovered TB206-001 monoclonal antibody antagonist to adenosine A2A receptor, which has a nanomolar binding of hA2AR-overexpressing HEK293 cells and has cross-reactivity with mouse and cynomolgus A2AR. However, it does not cross-react with human A1, A2B, or A3 receptors. In the in vivo model, the antibody has tumor-suppressing activity in colon tumor-bearing HuCD34-NCG mice. The data are exciting and can be a basis for the manuscript: some technical issues of some results and other issues are described below.

1. Although the author has claimed the antagonist activates T-cells in TME. However, natural killer cells also express A2AR and also produce IFN-γ. Does the antagonist affect NK cells?
2. T cell activation could further be assessed by (1) proliferation and (2) up-regulation of activation markers (e.g., IL2RA/CD25).
3. In panel 4 of the results, the author should provide the image of tumor volume.
4. All the bar diagrams used in the study should be modified to dot plots in bar diagrams to increase the transparency related to the independent biological replicate experiments.
5. The authors should discuss more about the immunological aspect of the diseases and cite the relevant manuscripts that would make the manuscript better flow and better suited for publication.
